# Machine Learning-Based Epileptic Seizure Detection Methods Using Wavelet and EMD-Based Decomposition Techniques: A Review

**DOI:** 10.3390/s21248485

**Published:** 2021-12-20

**Authors:** Rabindra Gandhi Thangarajoo, Mamun Bin Ibne Reaz, Geetika Srivastava, Fahmida Haque, Sawal Hamid Md Ali, Ahmad Ashrif A. Bakar, Mohammad Arif Sobhan Bhuiyan

**Affiliations:** 1 Department of Electrical, Electronic and System Engineering, Universiti Kebangsaan Malaysia, Bangi 43600, Malaysia; P108081@siswa.ukm.edu.my (R.G.T.); mamun@ukm.edu.my (M.B.I.R.); p97694@siswa.ukm.edu.my (F.H.); sawal@ukm.edu.my (S.H.M.A.); ashrif@ukm.edu.my (A.A.A.B.); 2 Department of Physics and Electronics, Dr. Ram Manohar Lohia Avadh University, Ayodhya 224001, India; gsrivastava@rmlau.ac.in; 3 Department of Electrical and Electronics Engineering, Xiamen University Malaysia, Bandar Sunsuria, Sepang 43900, Selangor, Malaysia

**Keywords:** electroencephalogram, wavelet, empirical mode decomposition, random forest, support vector machine

## Abstract

Epileptic seizures are temporary episodes of convulsions, where approximately 70 percent of the diagnosed population can successfully manage their condition with proper medication and lead a normal life. Over 50 million people worldwide are affected by some form of epileptic seizures, and their accurate detection can help millions in the proper management of this condition. Increasing research in machine learning has made a great impact on biomedical signal processing and especially in electroencephalogram (EEG) data analysis. The availability of various feature extraction techniques and classification methods makes it difficult to choose the most suitable combination for resource-efficient and correct detection. This paper intends to review the relevant studies of wavelet and empirical mode decomposition-based feature extraction techniques used for seizure detection in epileptic EEG data. The articles were chosen for review based on their Journal Citation Report, feature selection methods, and classifiers used. The high-dimensional EEG data falls under the category of ‘3N’ biosignals—nonstationary, nonlinear, and noisy; hence, two popular classifiers, namely random forest and support vector machine, were taken for review, as they are capable of handling high-dimensional data and have a low risk of over-fitting. The main metrics used are sensitivity, specificity, and accuracy; hence, some papers reviewed were excluded due to insufficient metrics. To evaluate the overall performances of the reviewed papers, a simple mean value of all metrics was used. This review indicates that the system that used a Stockwell transform wavelet variant as a feature extractor and SVM classifiers led to a potentially better result.

## 1. Introduction

Epilepsy is a neurological disorder that affects nearly 50 million people worldwide [1]. The detection of the onset of an epileptic seizure is an important parameter in reducing the work hazards and other related risks for seizure patients by allowing the relevant drugs to be administered quickly. The detection and diagnosis of epileptic seizures often require that the patient’s electroencephalogram (EEG) signals are monitored for a long duration of time. However, the manual visual inspection process for these long-duration signals, in addition to physiological and non-physiological artifacts, makes interpretation a daunting and challenging task. Automated systems are available that utilize machine learning algorithms to learn EEG patterns to identify brain neuronal activities that could lead to potential seizures. These include events of interest from background patterns, which could be based on time, frequency, time-frequency, or nonlinear methods [2].

**Figure 1:**
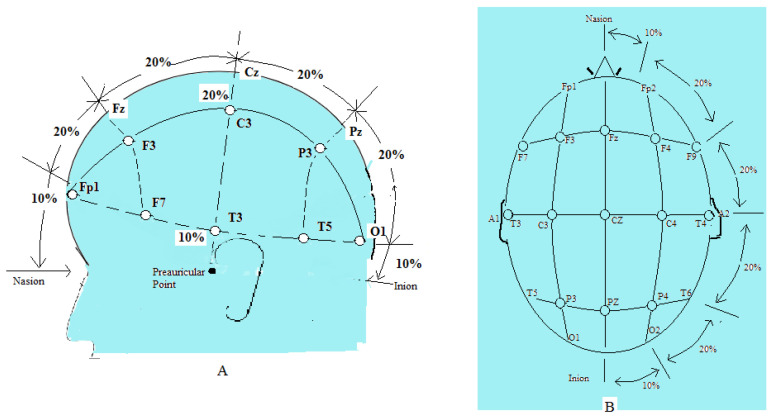
The international 10–20 system of the electrode [4]. Seen from (**A**) left and (**B**) above the head. A: earlobe, C: central, Pg: nasopharyngeal, P: parietal, F: frontal, Fp: frontal polar, O: occipital.

A part of the ongoing research in the field of EEG-based epilepsy detection using machine learning focuses on the training of recognizable seizure activity by the machine learning model and its deployment thereafter. For this, the system uses non-invasive brain electrodes placed on the human brain. One common method reported for detecting seizures is based on the recognition of relevant changes in power and frequencies and the emergence or disappearance of signal modes [3]. This is needed to identify if the seizure type is a general or focal seizure. Thus, it is important to give proper placement of the brain electrode on the patient. The papers used in this study are based on the 10–20 system of electrode placement for EEG recording, as shown in Figure 1 [4]. With this, seizure detection using machine learning does not only recognize seizure events, but also their locations.

**Figure 2:**
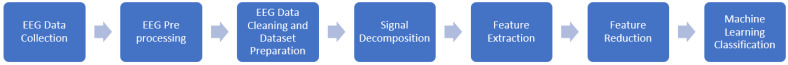
The process of epileptic seizure data classification.

Figure 2 shows the basic process of EEG-based epileptic seizure data classification. Seven of the studies [1,5,6,7,8,9,10] discussed in this paper followed the block diagram in Figure 2, where the papers [1,5,8,9,10] are used for comparison. Another six studies [10,11,12,13,14,15] did not use the feature reduction block of Figure 2. Papers [12,16,17] had pre-processing features in the extraction block, in which decomposed signals were pre-processed first before the features were extracted. Papers [18,19,20,21] mentioned here did not have a signal decomposition block. In addition, one paper [21] classified its signals using segmentation statistics and not machine learning classifiers.

In this paper, 19 articles are reviewed and compared for their performance, but only 10 of them provide the full set of sensitivity, specificity, and accuracy. Thus, these metrics are used for comparative study in this review. Table A1 in Appendix A has been provided for further information on all papers used in this review.

### 1.1. EEG Databases and EEG Recording Techniques

This study involved the investigation of several databases used in extracting EEG information. Ten of the studies used the database from the Epilepsy Center of the Bonn University Hospital of Freiburg in Germany. Five studies used the CHB MIT database, available online: https://paperswithcode.com/dataset/chb-mit (accessed on 1 December 2021). The rest of the databases were used in only one of the studies, including the Toronto Western Hospital Epilepsy Monitoring Unit, the University Hospital of Rennes in France, the Mount Sinai Epilepsy Center, Xi’an Jiaotong University, and a dataset from the Mayo Clinic (website). Table A2 has been provided in Appendix B to reflect the different techniques involved in EEG recordings.

The CHB-MIT database contains non-invasive extracranial scalp EEG data collected from 24 patients with 9–42 recordings for each patient. Each recording was collected at a sampling rate of 256 Hz and lasted around 1 h. All 24 patients provided their age and gender [1,7,10,12,21].

The Epilepsy Center of the Bonn University Hospital has a database that consists of five subsets of EEG signals, denoted as Set Z, O, N, F, and S. Each one of the subsets contains 100 single-channel EEG recordings, where each segment in a set has a 23.6 s duration collected at a sampling frequency of 173.61 Hz. [6,9,11,12,13,14,16,17,20,22].

The Toronto Western Hospital Epilepsy Monitoring Unit patient recordings were collected from 12 subjects. All of them underwent pre-surgical evaluation. Patients were selected based on the availability of interictal and clinical seizures records, which are separated on an hourly basis. A total of 23 seizure events were recorded for six patients for the training phase, while the testing phase was executed for 12 patients with a record of 33 seizure events [5].

The Mount Sinai Epilepsy Center dataset contained 86 scalp EEG recordings. All of the recordings are continuous EEG studies that were obtained from 28 patients with epilepsy. The recordings were made with 22 inputs and a 256 Hz sampling rate. The monitoring duration lasted anywhere between 2 and 8 days [7].

The Institutional Review Boards of Xi’an Jiaotong University provided a dataset of ten patients with medically intractable partial epilepsy. The recorded EEG signals were sampled at 200 Hz. They used 19 channel electrodes that were placed according to the standard international 10–20 system [8].

The dataset from the Neurology Department of the University Hospital of Rennes in France contains intracranial EEG (iEEG) signal recordings. They come from five patients suffering from drug-resistant epilepsy. They used a recording system with a sampling rate of 2048 Hz [15].

The dataset from the Mayo Clinic website was obtained from a continuous stream of iEEG recordings, available at the website (www.kaggle.com/c/seizure-prediction/data, accessed on 1 December 2021 ). This is the only study that used measurements from six dogs. They were recorded from 16 channels of raw iEEG data sampled at 400 Hz. All dogs that were studied suffered at least seven seizure episodes on record, where only one had just three leading seizures [19].

### 1.2. EEG Decomposition Methods

**Figure 3:**
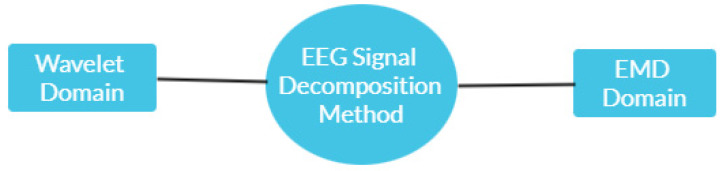
Seizure decomposition method.

The raw EEG signal is based on a time series, time-varying signal that contains components that vary in amplitude, frequency, and phase along the time axis. To process such signals for feature extraction or classification, they need to be decomposed into their constituent amplitude and frequency components. Only then will the relevant feature vectors be extracted from the decomposed components. Any combinations of the three components mentioned here can be used for extraction. The two main decomposition methods discussed in this study use the many different variants of the wavelet transform and empirical mode decomposition (EMD). As shown in Figure 3, the wavelet is a time-frequency domain-based transformation, while the EMD is a time series-based transformation method.

The wavelet transform is an engineering solution for processing signals that are not constant in frequency. Frequency-based signals cannot be processed effectively by simple methods, such as fast Fourier transform, as it gives the frequency content information only if the signal is stationary, which seldom occurs in nature. The next improvement was the short-time Fourier transform, which gives frequency information as well as information on the time at which the signal’s frequency occurs. The drawback of this method is that the windowing used to extract this information is not robust. This creates a resolution problem. Thus, with the next improvement comes the wavelet transform. It is able to give information on both the signal’s frequency and magnitude. It can also provide the timing information for the occurrences of the signal’s events.

Wavelet [23] was used in six studies in this review for time-series events, and the analysis usually involves a spectrogram. The following formula illustrates a basic use of the wavelet transform [5]:

(1) $$W(s,\tau )=\int_{t}x\left(t\right)*\phi_{s,t}^{*}\left(t\right)dt\;\mathrm{where}\;\phi_{s,t}^{*}\left(t\right)=\frac{1}{\sqrt{s}}\phi_{o}\left(\frac{t-\tau }{s}\right)$$

The equation on the left is the wavelet function and the equation on the right is called the mother wavelet (Morlet in the case of [6]), which is convolved with the signal to obtain the transformed time-frequency analytical signal. The variants used in these studies were the empirical wavelet transform [1], continuous wavelet transform (CWT) with Morlet [5], tunable Q wavelet transform [6], and wavelet decomposition [18].

**Figure 4:**
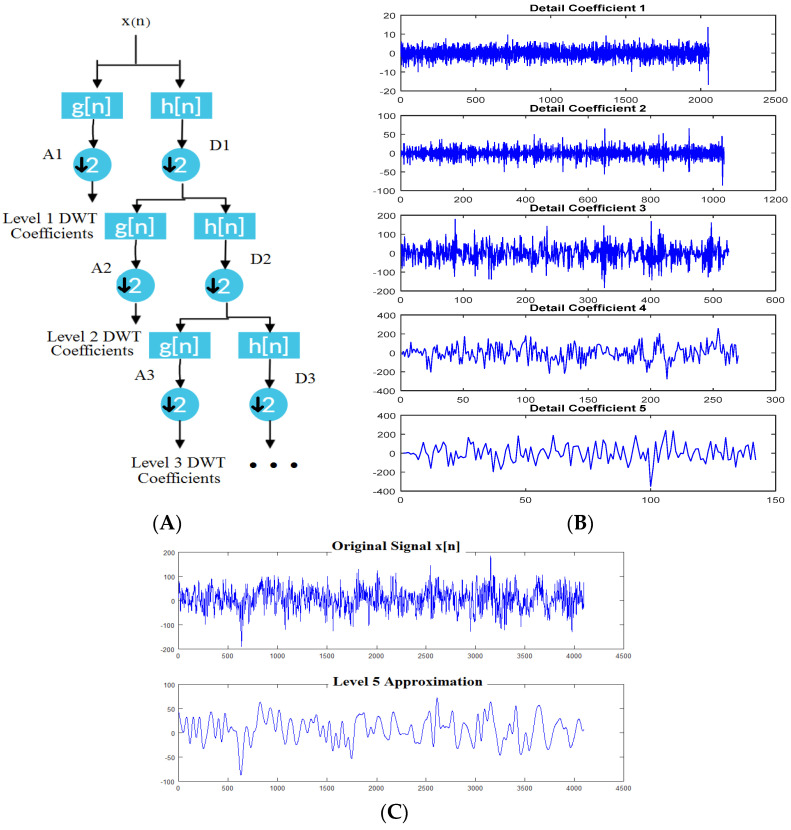
The wavelet decomposition method used to process a signal x(n). (**A**) Decomposition of a signal into its approximate (g(n)) and detail (h(n)) coefficients. (**B**) The signal decomposed into its five level detail coefficients. (**C**) Example of what a fifth level decomposition looks like.

Figure 4 is an example of a discrete wavelet transform output obtained from Matlab^®^. It was obtained by using a sample signal supplied by Matlab 6.1 and can be decomposed by anyone with access to the software. Figure 4A shows how a signal is decomposed into its high-pass output of approximate coefficients and its low-pass output of detail coefficients. At every stage, both filter outputs are downsampled by a factor of two before going through the process again on several levels. Figure 4B shows such a process, where the output of five levels of detail coefficients is obtained, which is relevant for decomposition. Figure 4C shows a comparison of the original signal and the signal after going through the fifth level of filtering. All the detail coefficients are further used for frequency analysis.

Table 1 is a list of the types of wavelets used in the first six papers, reviewed on the basis of the wavelet decomposition methods used. The other decomposition method used was the empirical mode decomposition variant [11,16,17]. This is an algorithm that is data-dependent and functions to produce a group of intrinsic mode functions (IMFs) for a time-series signal. The IMFs can characterize the frequencies of the time-series signal by decomposing the signal into its constituent frequencies, from the highest to the lowest. The instantaneous time-series frequencies can then be obtained from each IMF. A popular method to do this is to process them using Hilbert transform, which separates each IMF into its complex components and transforms it for the frequency information.

The formula for EMD is very intuitional and there is diversity in terms of its variants. The general equation is:

(2) $$x(t)=\sum_{m=1}^{M}C_{m}\left(t\right)+r\left(t\right),\mathrm{where}\;m=1,\;2,\;...,\;M,$$

where M is the number of IMFs, and C_m_(t) and r(t) are the mth IMF and the decomposition residue, respectively. The complete ensemble EMD with adaptive noise [11] and the variational mode decomposition [16] are referenced in this study.

**Figure 5:**
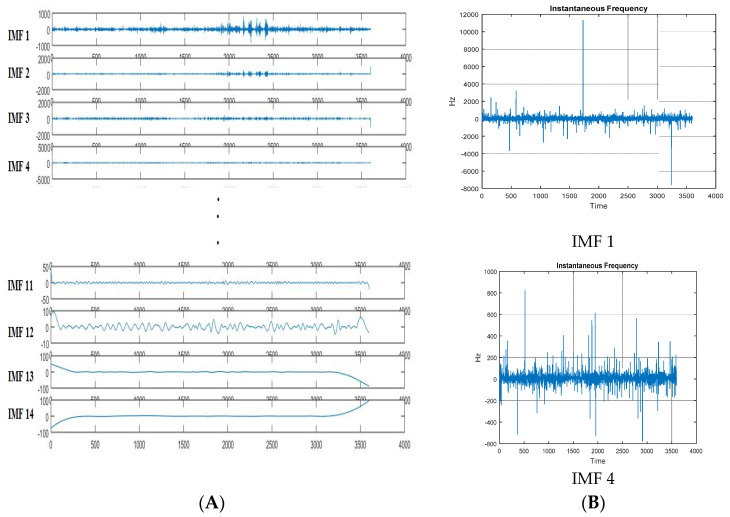
The process of signal decomposition using empirical mode decomposition. (**A**) EEG signal of one electrode decomposed into 14 separate intrinsic mode functions, only the first and last four are shown here for clarity. (**B**) Process of Hilbert transform used to obtain each IMF’s instantaneous frequency information; the example shown here is only for IMF1 and IMF4. Signal was obtained from the CHB MIT database and was single-channel processed using Matlab 2015a.

Figure 5 shows the decomposed IMF of a sample EEG signal of one electrode. After obtaining the IMF, its instantaneous frequency information is obtained by using the Hilbert transform, as shown in Figure 5B. The amplitude or frequency changes obtained here can be used for further feature extraction.

### 1.3. EEG Pre-Processing Techniques and EEG Artifacts/Data Cleaning

**Figure A1:**
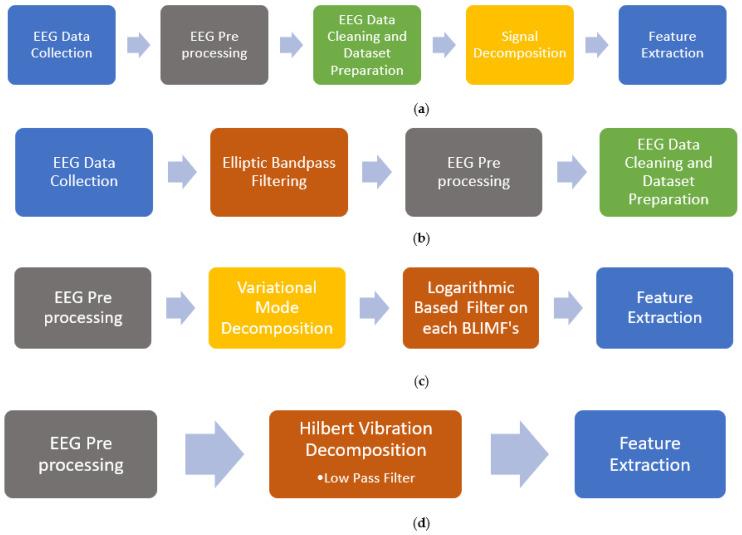
EEG pre-processing techniques and EEG artifacts/data cleaning used in this review. (**a**) The first five processes of epileptic seizure data classification as in Figure 2. (**b**) The epileptic filter used to bandpass the frequency from 0.5 Hz to 60 Hz in study [12]. (**c**) The logarithmic operation applied to each BLIMF after decomposition in study [16]. (**d**) The 4th order Butterworth low-pass filter embedded in the decomposition phase in study [17].

The process of decomposition breaks down a non-stationary signal into its separate frequency and amplitude components. However, some decomposition techniques produce output that needs pre-processing, as it cannot be used for extraction immediately. There are various reasons why this happens, as outlined in the studies of Section 2.2 [16,17] and Section 2.3 [12] which used pre-processing features. Figure A1 has been provided in Appendix C to highlight the different techniques used.

In [12], a pre-processing block was used to remove any brain pattern noise of the acquired EEG signal before it was treated by a decomposition process. This was performed using elliptic band-pass filters, which efficiently keep the signals to a frequency limit between 0.5 Hz and 60 Hz. Only then were the signals decomposed using discrete wavelet transform.

In [16], Zhang, Chen, and Li used the variational mode decomposition method. It functions to process the raw EEG signal and separate it into 15 sets of band limited intrinsic mode functions (BLIMFs). Since the amplitude fluctuates violently for each BLIMF, each BLIMF undergoes a mathematical operation (logarithmic in nature) to reduce the effects of volatility, then new BLIMFs are generated (after which they are known as nBLIMFs). The equation for the logarithmic operation can be stated as: nBLIMF = sgn(BLIMF) • log α(1 + |BLIMF|).

In [17], Mutlu used Hilbert vibration decomposition in his technique. However, for this study, the intended decomposition output consisted of symmetric quasi-harmonic oscillations where the signals of different modes, called mono-components, were embedded inside it. Three iterative processes were required for the mono-components to be computed. First, the mono-component with the largest energy was estimated for the first instantaneous frequency. Then, the signal was low-pass filtered by a 4th order Butterworth filter before synchronous detection was used to obtain the signal’s envelope. This preprocessing was completed so as to ensure a 4 Hz bandwidth between each mono-component. Finally, the estimated initial time series mono-component was subtracted before the extraction process could begin.

### 1.4. Feature Extraction Methods Used in This Review

Feature extraction methods are unique to all the studies. Though each study chose a novel feature for their papers, invariably all papers reviewed here will fall into three major extraction technique categories. The categories are based on energy, basic statistics, or a distribution/histogram. There is also one last sub-section that discusses papers that combine the techniques mentioned.

#### 1.4.1. Extraction Based on Energy

Parvez and Paul [18] first divided the EEG signals into smaller epochs before utilizing two major features. One was the undulated global feature (UGF) while the other was the undulated local feature (ULF). For the UGF, phase correlation was used to detect the relative variation between the current and successive epoch of EEG signals. The Fast Fourier Transform (FFT) was first applied to the signals and then followed up by phase correlation, which is determined using Inverse FFT (IFFT) and shift FFT functions. Detections were performed for ictal or non-ictal signals. All frequency components were found after the minimum mean square error (MSE) between both epochs was calculated and passed through the discrete cosine transform (DCT). Finally, the mean energy concentration ration (MECR) was calculated and used as the UGF. The undulated local feature (ULF) was extracted using the fluctuation and deviation of EEG signals within the epoch. A fluctuation function (*f*) was calculated for each shifted epoch. The epochs used were 10 s long with the amount of shifted sample at 128 samples. The final feature used was a cost function, which is called the energy function of cost function and deviation (ECFD).

Jrad et al. [15] decomposed their EEG signals using Gabor atoms that provided events of interest (EOI), consisting of four types of high-frequency oscillations (HFOs). From here, the features were extracted using an energy ratio called the Gabor root mean square (RMS) [16,17,18] and a temporal feature. The Gabor RMS is used to empirically compute a cumulative distribution function (CDF) of the entire signal. Its formula is shown in Equation (3) below, where c is the Gabor transform of raw iEEG signals and *w*_N_ is the sliding rectangular window of width N. An optimized threshold, λ, which denotes the percentile of the CDF, was set as a discriminator between the high-frequency oscillations and false alarms. Finally, five discriminant feature vectors were processed from the EOI to form vectors ‘ɤ’, ‘Hɤ’, ‘Rs’, ‘FRs’, and ‘ART/IES’ and to be processed by the classifier.

(3) $$\mathrm{Gabor}\;\mathrm{RMS}\;\mathrm{Energy}\;\varepsilon^{j}(t)=\sqrt{\frac{1}{N}\sum_{i}{\left(c\left(i;f_{0}^{\left(j\right)},\sigma^{\left(j\right)}\right)\right)}^{2}}\;wN_{\;}(i-t),$$

Kaleem, Guergachi, and Krishnan [10] provided four frequency bands (δ, θ, α, and β) for feature extraction. Each band contributed three features (12 features per channel per signal). The energy feature (Ei) was directly extracted from the decomposed band. The other features were processed using fast Fourier transform and extraction was completed from its frequency domain representation; these are the sparsity of amplitude spectrum (Sp$\hat{f}$_*i*_) and the sum of derivative of amplitude spectrum (D$\hat{j}i$). Both equations are shown in Equations (4) and (5), where M is the length of $\hat{f_{i}}$.

(4) $$\mathrm{Sparsity}\;\mathrm{of}\;\mathrm{Amplitude}\;\mathrm{Spectrum}\;\mathrm{Sp}{\hat{f}}_{i}\;=\frac{\sqrt{M\;}–\left(\sum_{m=1}^{M}\hat{f}i\left[m\right]\right)/\sqrt{\sum_{m=1}^{M}{\hat{f}}_{i}^{2}\left[m\right]}}{\sqrt{M}-1},$$

(5) $$\mathrm{Sum}\;\mathrm{of}\;\mathrm{Derivative}\;\mathrm{of}\;\mathrm{Amplitude}\;\mathrm{Spectrum}\;D\hat{j}i\;=\sum_{m=1}^{M-1}\hat{{\overset{´}{f}}_{i}}{\left[m\right]}^{2},$$

Goksu [22] used a much simpler energy-based extraction technique, which was supplied after the EEG signal was decomposed by wavelet packet decomposition. The three energy-based feature vectors used here were the log energy entropy, norm entropy, and energy, as shown in Equations (6)–(8) below.

(6) $$\mathrm{Log}\;\mathrm{Energy}\;\mathrm{Entropy}\;E=\sum_{n}\log\left(w_{j,k}^{n2}\right),$$

(7) $$\mathrm{Norm}\;\mathrm{Entropy}\;E=\sum_{n}{\left|w_{j,k}^{n}\right|}^{p},$$

(8) $$\mathrm{Energy}\;E\;=\sum_{n}\left(w_{j,k}^{n2}\right),$$

Tsiouris et al. [21] used the short-time Fourier transform (STFT) to extract the energy distribution of an EEG signal. Energy information from each channel was accumulated as a spectrum of the total brain activity distribution for each 1 h epoch. Finally, the energy contents of the δ, θ, and α frequency bands were separated. The focus was to detect high activity rhythms. However, this work did not use the machine learning method; instead, the next process of classification used the segment selection method.

Ali [17] used the Hilbert vibration decomposition (HVD) to extract the mono-components which had the necessary time-frequency information in feature extraction. This paper estimated the largest energy for the mono-component and its instantaneous frequency. Using synchronous detection, its envelope was obtained and was fed to the classifier. The estimated mono-component was then subtracted from the initial time series.

#### 1.4.2. Extraction Fully Based on Statistics

Basic statistics, for example, the mean, variance, standard deviation, and kurtosis [24] can be used in the extraction phase, and their formulae are provided below. Note that the standard deviation, σ, is just the square root of the variance, σ^2^. Additionally, the denominator for kurtosis, K, is the squared value of the variance [25]. These are the general equations that were used in the following four papers that used the statistical-based feature extraction technique.

(9) $$\mathrm{Mean}\;(µ)=\frac{1}{N}\sum_{t=1}^{N}x\left(t\right),$$

(10) $$\mathrm{Variance}\;(\sigma {)}^{2}=\frac{1}{N}\sum_{t=1}^{N}{\left(x\left(t\right)-{µ}_{x}\right)}^{2},$$

(11) $$\mathrm{Kurtosis}\;(k)=N\frac{\sum_{t=1}^{N}{\left(x\left(t\right)-{µ}_{x}\right)}^{4}}{\sum_{t=1}^{N}{\left({\left(x\left(t\right)-{µ}_{x}\right)}^{2}\right)}^{2}}-3,$$

Bhattacharyya and Pachori [1] used an information entropy-based method to firstly reduce the number of EEG signals to be processed based on amplitude fluctuation in EEG signal, before decomposing it into its sub-bands using empirically chosen wavelet transform. Each sub-band was extracted into their individual MODES which, when decomposed further, reveal their amplitude and frequency components. The individual instantaneous amplitude was joint and its feature was later extracted using a statistical process. The three features used here were the joint instantaneous amplitude (µ), mean monotonic absolute AM change (*v*), and variance of monotonic AM change (σ). This was be obtained for each MODE. Finally, each of these features was combined for each mode to form the joint feature vector before classification.

Zhang, Chen, and Li [16] used an auto-regression (AR)-based quadratic feature extraction process that uses heavy statistics. It relies on two parameters, which are a maximum likelihood estimation function and Burg’s method, designed to minimize the prediction error power. The AR model was used to calculate the AR reflection coefficient as shown in Equation (12), where *m* is the order of the AR model. This model was processed further by four other statistical-based criteria to optimize the AR model; these were the final prediction error (FPE) criterion, Akaike information criterion (AIC), Bayesian information criterion (BIC), and criterion autoregressive transfer (CAT). Since the AR coefficient data was diverse in terms of dimensions, another eight statistical parameters of the AR coefficients were extracted as secondary measures and fused together as feature vectors of the EEG sequence. These parameters were the energy, length, maximum, minimum, mean, variance, skewness, and kurtosis of the coefficients of the best AR model.

(12) $$\mathrm{AR}\;\mathrm{coefficients}\;a_{\hat{m},i}=\{\begin{array}{c} a_{m-\hat{1},i}+{\hat{k}}_{m}a_{m-\hat{1},m-i}^{*}\;,\;\;i=1,2,\ldots ,m-1\\ {\hat{k}}_{m},\;\;i=p \end{array}$$

Li, Chen, and Zhang [14] applied envelope analysis using Hilbert transform to their decomposed EEG signal to start the extraction process. The envelope contained valuable information on different scales. Extraction was very straightforward with the following statistical extraction methods, which were affected on each sub-band of the envelope spectrum. These were the mean, energy, standard deviation, and maximum value. Together, they form a 20-dimension vector.

Jia, Goparaju, and Song [11] made use of 2D and 3D phase state representation of EEG signals, which have been used in the past to measure the uncertainty of signals such as an epileptic seizure or non-seizure. A Euclidean distance function was used to measure the spread of data in the state space between adjacent points to calculate a growth curve. Finally, five spectral moment-based features were extracted from each IMF, including the spectral decrease, spectral centroid, spectral spread, spectral flatness, and spectral slope. All equations are as shown below.

(13) $$\mathrm{Spectral}\;\mathrm{decrease}\;SD=\frac{\sum_{q=1}^{M-1}\;\;\frac{1}{q\;\;}\left(\left|Y\left(q\right)-Y\left(0\right)\right|\right)}{\sum_{q=1}^{M-1}\;\;\left|(Y\left(q\right)\right|},$$

(14) $$\mathrm{Spectral}\;\mathrm{flatness}\;SF=\frac{\prod_{q=0}^{M-1}\;{\left|Y\left(q\right)\right|}^{\frac{1}{M}}}{\frac{1}{M}\sum_{q=0}^{M-1}\;\;\left|(Y\left(q\right)\right|},$$

(15) $$\mathrm{Spectral}\;\mathrm{centroid}\;SC=\frac{\sum_{q=0}^{M-1}q\;\;\left|(Y\left(q\right)\right|}{\sum_{q=0}^{M-1}\;\;\left|(Y\left(q\right)\right|},$$

(16) $$\mathrm{Spectral}\;\mathrm{spread}\;SS=\frac{\sum_{q=1}^{M-1}{\left(q-SC\right)}^{2}\;\left|Y\left(q\right)\right|}{\sum_{q=0}^{M-1}\;\;\left|(Y\left(q\right)\right|},$$

(17) $$\mathrm{Spectral}\;\mathrm{slope}\;SSL=\frac{M\sum_{q=0}^{M-1}fm\;\;\left|(Y\left(q\right)\right|-\sum_{q=0}^{M-1}fm\;\;\;.\;\;\sum_{q=0}^{M-1}\;\left|(Y\left(q\right)\right|}{\;\sum_{q=0}^{M-1}f_{m}^{2}\;-\;{\left(\sum_{q=0}^{M-1}\;\left|(Y\left(q\right)\right|\right)}^{2}},$$

#### 1.4.3. Extraction Based on Distribution and Histogram

Khan et al. [7] used three features: the pre-ictal, ictal, and interictal periods. A wavelet transform converted the brain signals into tensors, which were then fed into a convolutional neural network (CNN). This was used primarily to extract all three features by differentiating between them. With the true pre-ictal period being an unknown value, it must be guessed together with the best prediction horizon. Since this study’s main aim was to use the pre-ictal period to predict an oncoming seizure, it became an important parameter, (parameter l), which became the assumed pre-ictal length. Parameter l was used to label all periods before and after the seizure onset time, and its analysis included calculating two distributions from the periods. These were the interictal-only periods, which were approximated by a multivariate Gaussian with mean, µ0, and co-variance, ∑0. The other distribution was around a time point, t, also with mean, µ1, and co-variance, ∑1. A Kullback–Leibler divergence between both the distributions can detect shifts between them, which signals a 10 min warning before seizure onset.

Jacobs et al. [5] utilized the Morlet-based continuous wavelet transform that produced a spectrogram with a 2 s epoch for two frequency ranges: high frequency (fL) and low frequency (fH). The CWT supplies a complex-valued coefficient matrix that can derive an amplitude envelope time series A(t,fH) and phase time series φ (t,fL) used for feature extraction. The normalized mean of the amplitude envelope time series was calculated to represent a discrete probability density value, pj. This in turn was used to calculate a normalized entropy measure, called the index of cross-frequency coupling (IcFc), which measures the coupling between both time series, A, and phase series, φ. The IcFc gives a global measure of the cross frequency and results in a 131 × 91 co-modulogram. This results in a probability distribution which can eventually be used for classification purposes.

Jaiswal and Banka [20] made use of the unique patterns available in an EEG signal when there are signal abnormalities involved. They used each bit wide representation of the signal and processed them using the local neighbor descriptive pattern (LNDP) and one-dimensional local gradient pattern (1D-LGP). EEG signals have neighborhood relationship pattern properties, and their structures are maintained by the LNDP transformation. The 1D-LGP has also been previously shown to maintain this structure when it was used for EEG epileptic signal classification. These output of these transformations was condensed codes for classification. All transformation codes were then used to form a histogram, which forms the feature vector to be fed to the classifier.

Kalbkhani and Shayesteh [9] obtained five sub-bands from each EEG signal using the Stockwell transform. They then calculated the amplitude distribution of each sub-band, namely delta (δ), theta (θ), alpha (α), beta (β), and gamma (γ). A histogram that was normalized in relation to the histogram bins (Nb) was then formed. Each bin was considered as one feature for each sub-band. The variation and feature vector dimension of the EEG signals varied according to the histogram bin (Nb), thus an increase in Nb was followed up by feature reduction processes. 

#### 1.4.4. Extraction Based on Other Combination Techniques

Wang et al. [8] worked with normalized EEG segments, which were subtracted from the mean and divided by the variance. This was used to establish a multivariate auto regressive model (MVAR). A directed transfer function (DTF) algorithm was used to detect information flow intensities that signified brain activity. Thus, this technique is one that detects information entropy. The coefficients of the third order MVAR models were used here. The equation related to the DTF is shown below, where *H_ij_(f)* is the element in the *i*th row of *j*th column of the transfer matrix *H(f)*, and *h_i_(f)* is a column *I* of the transfer matrix *H(f)*.

(18) $${\mathrm{DTF}}_{\mathrm{ij}}(f)=\frac{Hij\left(f\right)}{\;\sqrt{h_{i}^{T}\left(f\right)h_{i}\left(f\right)}}\;,$$

Patidar and Panigrahi [6] used an entropy-based simple feature extractor. After decomposition, a low-pass and high-pass sub-band signal were obtained. They used the Kraskov entropy shown in Equation (19), which is an actual measurement of the Shannon entropy or the differential statistical entropy of signals. It uses the *k*-nearest neighbors’ sample with some distance measure, such as Euclidean distance, Hamming, or any other suitable distance measure.

(19) $$H_{k}=\phi (n)-\phi (k)\;+\;\log(C_{d})\;+\;\frac{d}{n}\;\sum_{i=1}^{n}\log\left(\xi_{i}^{k}\right)$$

Li, Chen, and Zhang [13] used a combination of entropy and permutation as feature extractors. After signal decomposition, the EEG signals were extracted using three methods. The Hurst exponent (H) is used to statistically measure the correlation between data points. Fractal dimension (FD) is a complexity measure tool designed to indicate how much fractal space it appears to fill. The last extractor is the permutation entropy (PE), which is effective for optimizing complex parameters and is widely used for processing large data sets.

Ibrahim, Djemal, and Alsuwailem [12] used a combination of entropy, statistics, and power to extract features. After decomposition by the discrete wavelet transform, they used the nonlinear methods Shannon entropy (SE) and largest Lyapunov exponent (LLE), which is a complexity measure of an EEG recording. In addition, they used the signal’s standard deviation and band power to extract relevant features.

Shiao et al. [19] is the sole paper in this review that used only power for feature extraction. After EEG signal decomposition into six Berger frequency bands, all outputs were squared to get the power for all 16 channels. This gave a 96-dimensional vector. This power was approximated by the use of the fast Fourier transform in each Berger frequency. Finally, the cross-channel correlation (XCORR) was calculated as a measure of similarity. This gave another 120-dimensional feature vector.

### 1.5. Machine Learning Algorithms Used for Review

The classification methods used in these studies rely heavily on machine learning techniques. Machine learning is a field of study that gives computers the ability to learn without being explicitly programmed [26]. The classification methods discussed in this review paper are mostly based on the random forest (RF) and support vector machine (SVM), as they are capable of handling high dimensional data [27] and have a low risk of overfitting [28].

Bhattacharyya and Pachori [1] used a seizure detection method using six classifiers for comparison. These were the functional tree, C4.5, random forest, Bayes-net, K-nearest neighbors, and naïve Bayes classifiers. The results indicated that the RF performed with the best accuracy at 99.41%. All classifications were applied using 10-fold cross-validation techniques.

In [5], the classification of a pre-clinical seizure state used a multi-stage state classifier (MSC), which contains 3 RF classifiers. Their system was assessed using a 5-fold receiver-operating characteristic (ROC) analysis, where they compared the performance under two conditions. For the system where the MSC received training, the accuracy was (95%), while for the system without training, it was (79.9%), thus proving that the RF classifier improves accuracy.

The study described in [29] is a good example of the simultaneous use of both random forest and SVM in a single paper. The authors used an optimized model, called the identification of sub-Golgi protein types (isGPT), to identify sub-Golgi protein types by use of equipment called the Golgi apparatus (GA). This application is found in medicine to prevent human disease, where any GA protein anomaly can result in congenital glycosylation disorders. After the feature extraction process, the random forest (RF) model was first used to give an important scoring to the features. It was then further used to rank the features based on these scores. Finally, the support vector machine (SVM) was used to classify the features of the top-ranked sub-Golgi proteins. Using this combination of machine learning techniques achieved an accuracy of 95.9%, 95.3%, and 95.4% for the leave one out cross-validation, independent testing, and 10-fold cross-validation, respectively.

Raghu and Sriraam [30] used several classifiers to categorize either focal or non-focal EEG signals, namely K-nearest neighbor (K-NN), SVM, adaptive boosting, and random forest. Cubic kernel-based SVM showed the highest accuracy at 96.1%. The other lower-performing classifiers were also based on SVM. However, RF still maintained the position of fourth-best classifier.

Based on these papers that used several classifiers, it is clear that in the cases where RF or SVM were used, they outperformed other machine learning techniques; hence, these classifiers appear ideal to be chosen for this review. The RF classifier was used by four studies, which were all used for result comparison, and support vector machine (SVM) variants were used by eight studies, where only five studies were used for result comparison.

**Figure 6:**
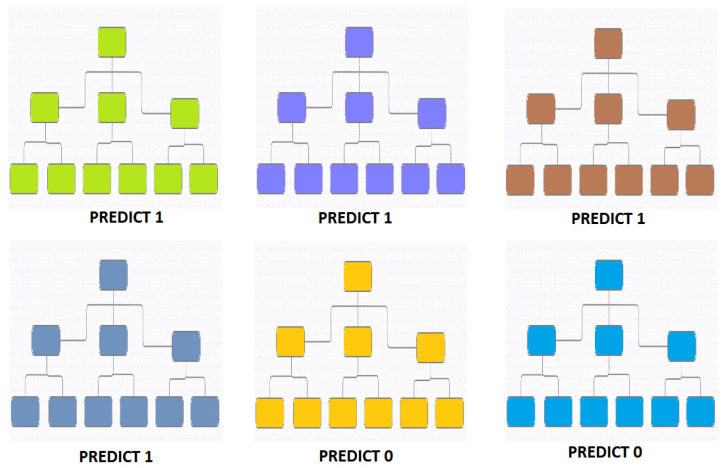
Ensemble decision tree structure that makes up a random forest classifier. The tally was four 1’s and two 0’s, thus resulting in prediction = 1.

Random forest (RF) is a fast machine learning classifier that is highly accurate and demonstrates resistance to noise artifacts [31]. It combines random feature selection and bagging. The random vector’s values, which are sampled separately, influence every tree in the forest. They also have an identical distribution to any other tree. RF consists of a massive number of decision trees. The trees select their separating features from the bootstrap training set Si, where ‘i’ represents the ith internal node. The classification and regression tree (CART) method is used to grow the trees without pruning. Studies that used the RF classifiers also utilized the empirical wavelet transform [1], CWT with Morlet [5], the CEEMDAN [11], and the variational mode decomposition [16]. Figure 6 shows one such RF technique, which is based on the ensemble decision tree. Out of all six trees, a majority of four predicted an output of 1. Thus, the prediction is taken as “1”.

**Figure 7:**
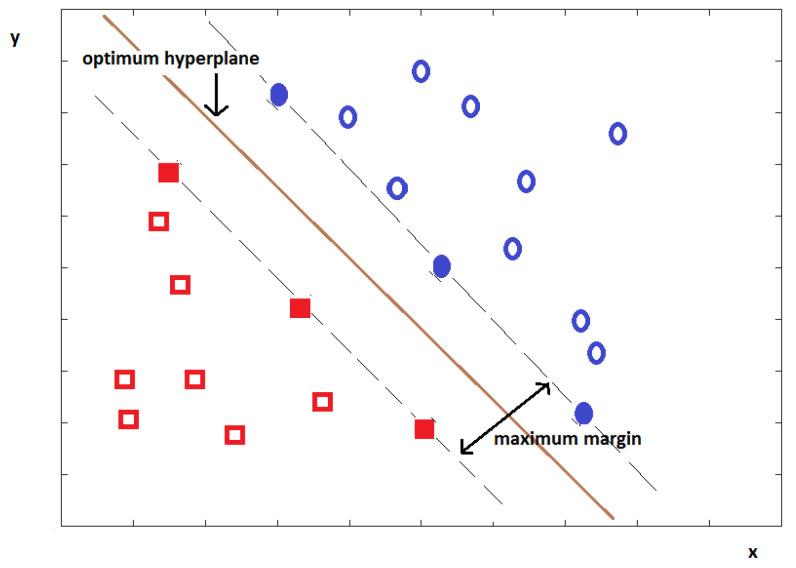
Hyperplane example for the support vector machine.

Support vector machine (SVM) is a machine learning technique that found its niche in applications that require regression-type analysis or classification. Another area that has seen its use is prediction-related activities, such as estimation or forecasting. SVM’s concept is the creation of a hyperplane that separates data as much as possible into two classes. The intention is to minimize the training set error by maximizing the boundary from the hyperplane. SVM works using kernel functions, among others, such as the RBF, polynomial or normalized polynomial kernel function [31]. The feature extraction methods most often used with the SVM are the three feature encoding [7], the undulated global and local feature [8], the DT-CWT [13], and the wavelet decomposition db4 method [18]. Figure 7 shows an example hyperplane of an SVM classifier. The hyperplane is calculated so as to separate the two classes of data (in blue and red) as much as possible.

## 2. State of the Art: ML-Based Epileptic Seizure Detection

Most of the 19 articles that will be reviewed here completely followed the classification process of Figure 2. However, eight papers followed the same process without the feature reduction block, and four of the papers did not use the signal decomposition block. This review takes the approach of comparing the performed of seven epileptic detection systems that use wavelet variants as a decomposition method. Further, the three system performance will also be reviewed based on the EMD variant decomposition method. These 10 systems will be reviewed again based on their machine learning classifiers. The performances of the four systems that used RF-based classifiers will be compared first, followed by another five systems that used the SVM classifiers. Section 2.1 will discuss the systems that are based on the wavelet decomposition systems, while Section 2.2 will discuss that with EMD as its decomposition system. Section 2.3 will discuss other systems which also use either the decomposition system or classifiers mentioned in Section 2.1 and Section 2.2, but could not be used in this review due to differences in the metrics used. Table A1 in Appendix A provides further information on all papers used in this review.

### 2.1. Wavelet-Based Decomposition Systems with Conventional Performance Metrics Parameters

Bhattacharyya and Pachori [1] employed the empirical wavelet transform (EWT), which from the Fourier point of view is a construction set of bandpass filters [32]. They utilized the Littlewood–Paley and Meyer wavelet for decomposition, using a novel technique of choosing only five EEG input channels for processing [33]. Their previous studies indicated that, in the long run, EEG detection only focuses on uni-variate analysis, which does not consider the cross-channel interdependence of the input’s multivariate data. Thus, their research minimized this input to five channels by using mutual information (MI), which measures similarity or interdependency among all channels. After decomposing these channels using EWT, they used the Hilbert transform to find the instantaneous amplitude and frequency information from the EEG signals. This information was used to form the multivariate time-frequency coefficients. The instantaneous amplitude information alone was used to derive three feature vectors which were processed by another feature processing stage. The vectors went through a moving average filter, which was used to enhance the magnitude corresponding to seizure segments. However, since this filter also enhances features in seizure-free segments and therefore introduces more false detection of epileptic seizures, all features were processed by the Hadamard transform to remove bias. In addition, this reduced over-fitting for the classifier’s input by enhancing seizure segments and reducing seizure-free segments. The database used in this article was from CHB-MIT and only 1.6% of the total EEG time belonged to seizure segments, which led to class imbalance problems. Three iterations of the synthetic minority oversampling technique (SMOTE) were used to correct the imbalance problem. The features were tested with RF, naïve Bayes, functional tree, k-NN classifiers, and Bayes-net. The best result came from the RF classifier using 10-fold cross-validation with a sensitivity of 97.91%, specificity of 99.57%, and accuracy of 99.41%.

In the research performed by Jacobs et al. [5], the focus was to detect seizures using features that can be generalized across patient datasets while still providing a low rate of false alarms and detecting seizures as early as possible. This is a challenge even though patient-specific algorithms have been used in combination with classifiers, including SVM, recurrent neural network, and logic-based algorithms. The authors used a Morlet continuous wave transform for signal decomposition and a novel method called cross-frequency coupling index (IcFc) for feature extraction [34]. The Tort modulation index was selected as the cross-frequency coupling CFC measure, as it assesses the coupling between an amplitude envelope-time series and an instantaneous phase-time series [35]. The IcFc produced a 131 × 91 co-modulogram which produced 11,921 feature vectors. Then, they were put through a binary threshold to reduce the feature vector while preserving the high-frequency contents above 60 Hz.

**Figure 8:**
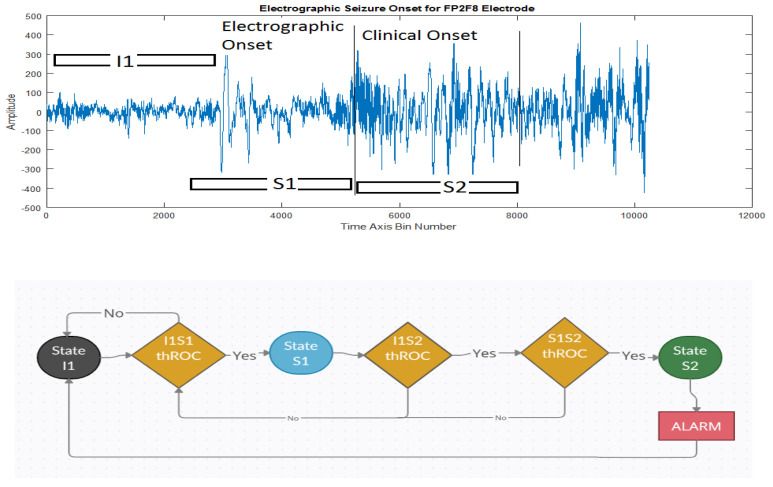
The 3RF classifier-based state machine. The th_roc_ value is determined at each stage of the state machine.

Figure 8 shows a multi-stage classifier, which is a state machine-based 3RF classifier with a basic logical decision threshold governing internal state transitions. It uses multi-iteration five-fold receiver operating characteristics (ROC) based on the cross-validation technique. The th_roc_ threshold’s exact value is determined from each RF classifier within the multi-stage classifier (MSC). The initial IcFc is first passed through the state machine at state 1 (S1). Each state analyzes the input based on a set th_roc_ and the ROC. For each iteration where the alarm is activated, the state is reset, and a new th_roc_ value is applied to all the states. During this training phase, after parameter optimization, the th_cfc_ value was found to be in the 94th, 95^th^, and 53rd quantiles for the I1S1, I1S2, and S1S2, respectively, while the new calculated IcFc obtained from the new th_roc_ value was used for thresholding. This reduces the feature vectors for the next iteration of MSC classification.

Using this system, the authors reported a sensitivity of 87.9%, both specificity and accuracy of 82.4%, and area-under-the-ROC curve (AUC) of 93.4%. In addition, the alarm produced was 45 s to 16 s in advance of clinical seizure onset across seizures from the 12 patients.

**Figure 9:**
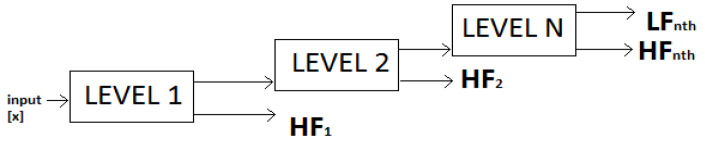
TQWT based N-level decomposition adapted from [6].

Patidar and Panigrahi [6] used a multi-stage tunable Q wavelet transform-based decomposition (TQWD) technique using the Daubechies filter with two vanishing moments. Their epileptic seizure analysis strategy was based on the use of linear prediction and fractional linear prediction methods [36]. The construction of Tunable Q-Factor Wavelet Transform (TQWT) filters is easier to implement in the frequency domain since they are based on non-rational transfer functions. The block diagram of the decomposition filter is reproduced in Figure 9. Here, two-band filter banks were attached repeatedly to the low-pass sub-bands signals. At each decomposition level, the input s[n] was converted into its low-pass and high-pass sub-band. Each filter was used by the TQWT, which is an empirically-chosen power complementary function with 2π periodic timing. They were selected as the frequency response for the Daubechies filter that had two vanishing moments.

For the feature extraction process, the novel Kraskov entropy measures were used since they can characterize non-linearities. By using the k-nearest neighbor’s sample with some distance, Kraskov entropy measures the Shannon entropy, or differential statistical entropy, of the signals. Distance measures that can be used are the Euclidean distance, Hamming, etc. [37]. This contributes to a probability distribution function used for feature extraction. Before being put through the classification process, the proposed feature set’s performance in discriminating seizure and seizure-free segments had to be evaluated by applying the Kruskal–Wallis statistical test. The LS-SVM with RBF kernel functions using 10-fold cross-validation resulted in a sensitivity of 97.00%, specificity of 99.00%, accuracy of 97.75%, and Matthew’s correlation coefficient of 96.00%.

Wang et al. [8] used the level 5 Daubechies order 4 wavelet-based decomposition followed by a novel wavelet directed transfer function technique for feature extraction. Their research aimed to solve the imbalance problem with EEG ictal signal lengths, which are less than interictal signals and can undermine detection performance and produce low selectivity. Furthermore, the large number of interictal EEG segments can also be mistakenly identified as ictal segments. Their approach was to use coefficients of multivariate auto regressive models [38], which in turn were used by the directed transfer function algorithm for feature extraction [39]. These features were used to measure the intensity flow between two channels. This produced a 19 × 19 feature matrix per segment; however, it was reduced to a 19 × 1 matrix per segment before being processed by the classifier. Using an RBF-SVM-based classifier with five-fold cross-validation, this method resulted in high accuracy with a value of 99.4%, a selectivity of 91.1%, sensitivity of 92.1%, specificity of 99.5%, and a detection rate of 95.8%.

Kalbkhani and Shayesteh [9] used the Stockwell transform (ST) to find frequency information in five sub-bands. The normal transforms, such as STFT or DWT transforms, cannot expose the amplitude content associated with each frequency, which is why the ST was used [40]. At lower frequencies, ST provides excellent time resolution, while for higher frequencies it gives high time resolution. There is also no need for any digital filter to find all the frequency components in the time-frequency domain. The author’s investigation of the ST EEG signal’s amplitude distributions in multiple sub-bands revealed five feature vectors, one for each sub-band obtained.

Earlier, the normal PCA [41] was used to reduce the dimensions of data without much loss of information. However, since PCA is linear, it is not suitable for processing EEG signals. It is said to have a complicated structure of higher-dimensional features and cannot represent the nonlinear relationship between features; thus, it is disadvantageous if used for feature reduction processes [42]. This paper used kernel principal component analysis (KPCA) to achieve feature reduction. Using the non-linear kernel principal component analysis method, the author’s extracted the information component from the feature vectors and used the nearest neighbor classifier with five-fold cross-validation. This paper used different dissimilarity distance measures between the sample test set and the data set for training before classification. The training sample class, with a minimum dissimilarity distance from the test set, had the test sample assigned to it. The author’s reported their performance results for three cases as follows: For the case of healthy signals, the sensitivity was 99.61%, the specificity was 99.83%, and the accuracy was 99.73%. In the case of interictal signals, the sensitivity was 99.53%, the specificity was 99.54%, and the accuracy was 99.32%. Finally, for ictal signals, the sensitivity was 99.42%, the specificity was 99.89%, and the accuracy was 99.73%. The ictal values are those used for the comparison between reviewed studies in this paper.

Kaleem, Guergachi, and Krishnan [10] employed a wavelet decomposition method using a level 5 Daubechies db6 mother wavelet with six vanishing moments [43]. Their investigation focused on the significant mixing of the EEG seizure/non-seizure states for epilepsy patients, since obtaining multiple recordings of multi-channel scalp EEG data is a challenging task [44]. This method did not require any feature processing for obtaining the seizure detection results. The authors used a three feature extraction technique, where one feature was the energy (Ei) directly contributed by the decomposed components. The other two features were extracted using the FFT from the decomposed components’ frequency domain representation. During the classification phase, there were two approaches used. In the first approach, seizure detection results were obtained by averaging all 23 channel classification results for each patient. The second approach involved choosing the channel with the highest value of the receiver operating curve (AUC) for each patient. This was achieved by using the area under the AUC of each channel as a performance measure. The second approach is novel since, for each patient, only one channel was used for seizure detection. This was achieved without having to fuse multiple channels post-classification or having to select the channel’s pre-classification results. A Student’s t-test was also designed as a feature ranking scheme used for further feature reduction. The study reported a sensitivity of 99.4%, specificity of 99.4%, and accuracy of 99.6%, with k-NN and SVM classifiers.

Li, Chen, and Zhang [13] tried to solve the unreal frequency component problem that occurs due to alternate sampling, where the traditional wavelet transforms cause halfway band separation. The disadvantage here is that it limits the information extraction capability of the features. The authors used the dual-tree complex wavelet transform (DT-CWT) because of its ability to use a dual-tree of wavelet filters and obtain the real and imaginary parts by generating complex coefficients. Features were extracted using the secondary method of the fractal dimension (FD), Hurst exponent (H), and permutation entropy (PE). The Wilcoxon test was used to test significance. The best result came from the support vector machine (SVM) near-symmetric 13/19 tap filters (NS 13/19) and Q-shift 14/14 tap filters (QS 14/14) classifiers. The authors reported an accuracy of 98%, sensitivity of 98%, and specificity of 100%.

### 2.2. EMD-Based Decomposition Systems with Conventional Performance Metrics Parameters

Jia et al. [11] used the complete ensemble empirical mode decomposition with adaptive noise (CEEMDAN) for extracting features from EEG signals [45]. Their study focused on the apparent mode mixing problem in EMD and wavelet-based decomposition techniques. CEEMDAN provides a better spectral separation of the modes and also permits accurate reconstruction using the IMFs and their residuals. The authors’ novel technique involved the use of the growth curve of the data in two-dimensional and three-dimensional phase space representation (PSR) to extract five spectral moment-based features [46]. The probability density function (PDF) of the symmetric normal inverse Gaussian (NIG) was used to fit the distribution of the data in the growth curve. After using an RF classifier with 10-fold cross-validation and a Kruskal–Wallis ANOVA to test significance, the authors reported their results for two conditions. One condition used sets S and (F, N), where the accuracy was 99%, the sensitivity was 99.5%, and the specificity was 100%. The other condition was for sets S and (F), where the accuracy was 98%, the sensitivity was 100%, and the specificity was 99%.

Zhang, Chen, and Li [16] used variational mode decomposition (VMD) which outputs band-limited intrinsic mode functions (BLIMFs). They found that, unlike STFT and WT, the EMD that is a data-dependent time-frequency analysis algorithm is able to recursively decompose an arbitrary signal into a series of subcomponents [47]. These are the intrinsic mode functions (IMFs). However, the IMFs are highly dependent on the methods of extrema point finding, the interpolation of extrema points into carrier envelopes, and the stopping criteria imposed. In addition, the resulting IMFs have mode mixing problems and limited mathematical understanding, which are the greatest challenges of EMD [48]. VMD can decompose a multi-component signal into a number of band-limited intrinsic mode functions (BLIMFs) non-recursively and synchronously [49]. VMD focuses on the variation problem and the solution of using augmented Lagrangian. The main strengths of VMD are that it possesses a more convincing mathematical theory and a rigorous derivation process, and it has the capacity to separate two harmonic signals with similar frequencies. After the process of decomposition using BLIMFs, a base-α logarithmic operation was imposed on each BLIMF to reduce fluctuation. Four different criteria were used to estimate the optimal autoregressive (AR) order, followed by calculation of the coefficients of the optimal AR model for the logarithmic scale of BLIMFs. The autoregression (AR)-based quadratic feature extraction was used together with a secondary measure to yield eight statistical parameters of AR coefficients, which were fused together as feature vectors of EEG sequence. The AR models were built using Burg’s method. A random forest classifier with 10-fold cross-validation was used with a resulting accuracy of 97.352%.

Mutlu [17] employed the Hilbert vibration decomposition (HVD) for EEG signal processing. The author investigated disadvantages involving EMD for EEG signals, specifically how it extracts IMFs only from a wide-band time series due to its lower frequency resolution. EMD is also computationally expensive [50]. HVD was used as it is effective at decomposing both narrow-band and wide-band signals. It can also detect signal components oscillating at desired or specified frequencies. Hilbert transform was used to obtain the analytic signal and instantaneous frequency for the decomposition of signals consisting of symmetric quasi-harmonic oscillations, using three iterative steps for the computation of mono-components [51]. These were signals that had distinctive time-varying amplitudes and instantaneous frequencies from non-stationary signals. First, the instantaneous frequency of the mono-component with the largest energy was estimated. Then, the signal’s envelope was obtained using synchronous detection. Finally, the estimated mono-component was subtracted from the initial time series. The HVD was used to construct an extracted feature set. Using a fourth-order Butterworth low-pass filter iteratively with a bandwidth of 4 Hz, each mono-component was ensured to have a maximum bandwidth of 4 Hz, thus extracting the EEG sub-band components. After using an LS-SVM classifier with a Mann–Whitney U test at a 5% significance level, the resulting accuracy from the RBF kernel function was 97.33% to 97.66%.

### 2.3. Wavelet-Based Detection Systems with Other Performance Metrics Parameters

The following section contains seizure detection systems that also used a wavelet-based decomposition method; however, they could not be used in this review since they have different metrics. However, their results are still interesting for further interpretation.

Khan et al. [7] presented a robust baseline model that did not have too many false-positive results. The decomposition technique used the continuous wavelet transform with the Mexican mother wavelet, which was applied to each EEG channel to yield tensors of wavelet coefficients in three modes (time, scale, and channel) [52]. Feature extraction was performed using a convolutional neural network. After processing the features using the SMOTE technique to solve the balance problem, the training was carried out using a deep-CNN classifier [53]. The classifier was trained with the cross-entropy loss function over three classes. The results were shown for two datasets: The Mount Sinai Medical Center dataset showed a pre-ictal length of 8 min and a false positive rate (FPr) of 0.128/h. The CHB MIT dataset gave a pre-ictal length of 6 min and a FPr of 0.147/hr.

Ibrahim, Djemal, and Alsuwailem [12] researched the diagnosis epilepsy and autism spectrum disorder (ASD) by investigating the different feature extraction and EEG classification techniques. They used a six-level DWT using Db4 EEG signal decomposition used in conjunction with largest Lyapunov exponent (Rosenstein’s algorithm) and Shannon entropy. They then combined the statistical method of standard deviation (SD) and band power (BP) for feature extraction. The cross-correlation approach was also used for extraction, where it was used to determine how well EEG channels were synchronized with each other. Altogether, six synchronization values formed the feature vector obtained. The classifiers they used were as follows: ANN with a log-sigmoid transfer function, containing one input layer, a five nodes hidden layer, and a soft-max normalized exponential transfer function output layer; the K-NN classifier used with majority voting; and finally, the linear SVM and LDA. The authors used a 10-fold cross-validation and obtained a result of 100% accuracy

Li, Chen, and Zhang [14] introduced a novel method of using the wavelet-based envelope analysis with a neural network ensemble to reveal hard-to-detect yet critical changes in the EEG signal. They used a level 5 Daubechies 4th order discrete wavelet transform that they configured with envelope analysis demodulated with Hilbert transform (HT) as feature extractors [54]. The characteristic features, which contain both valuable envelope and multi-scale information, were extracted from the envelope curves of the sub-bands. Then, 20-dimensional vectors were extracted. The authors used a neural network ensemble composed of three groups of networks, with five sub-nets in each group. They reported the accuracy of the system at 98.78%.

Jrad et al., [15] used the diversity of high-frequency oscillations (HFOs) in EEG signals to design a versatile detector [55]. They used a novel method that employed the convolution of the Gabor atom function with Fourier transform. This later decomposes into event of interest (EOI) signals, which are high-frequency oscillations. Since the energy ratio called the Gabor RMS feature and the temporal feature exist, their combination was used for the extraction of feature vectors. A radial basis function support vector machine (RBF-SVM) with five-fold cross-validation was used as a classifier. The EOIs provided the discriminant features and were used as the inputs to the classifier. There are four different types of HFOs. The classifier was used to classify detected events and label them as a HFO or a high-frequency artifact (ART/IES). The result was specified for ripples signal (Rs) and fast ripples (FRs). The sensitivity was 91.7% for Rs and 72.8% for FRs, while Sp was 73.8% for Rs and 93.3% for FRs.

Goksu [22] used the wavelet packet analysis method for the decomposition of EEG signals (WPD). WPD has better high-frequency resolution than wavelet analysis and better time representation than Fourier analysis. The classifier used was the multilayer perceptron (MLP) with back propagation learning. The input and output layers of the MLP used linear activation functions, while the hidden layer(s) used the hyperbolic tangent sigmoid activation function. The reported accuracy was 100% for the following sets of input, in the case where they used energy, WPD, log energy and norm entropy on normal vs. interictal vs. ictal signals. The authors also reported 100% accuracy in the case where they used WPD and norm entropy on the non-seizure vs. ictal signals.

### 2.4. Other Statistical and Segmentation-Based Detection Systems

The following studies present a good example of how detection systems can be designed without a decomposition method, but instead rely on statistical measures for feature extraction and classification.

Parvez and Paul [18] aimed to achieve a good balance between better accuracy for advanced prediction and a low false positive rate. They used a novel undulated global feature (UGF) from a different epoch and an undulated local feature (ULF) from within the same epoch. The UGF extraction process involved the EEG signal being divided into small epochs and the relative change being estimated between the current and successive epoch using phase correlation [56]. This detects signal type change for the ictal/non-ictal period. The UGF was used to calculate the mean energy concentration ratio (MECR). Meanwhile, the ULF extraction involved calculations using a 10 s epoch with 128 shifted samples and the fluctuation function (f) for each shifted epoch. Then, a cost function was calculated, which became the energy cost function of fluctuation and deviation (ECFD) [57]. Both MECR and ECFD were used as feature vectors for classification. The LS-SVM classifier with 10-fold cross-validation was coupled with a windowing regularization technique. A high prediction accuracy of 95.4% with a low FPr = 0.36/h was reported.

Shiao et al. [19] designed a new SVM-based system for seizure prediction, with the aim of choosing design choices and performance metrics that are closely correlated with clinical objectives [58]. They extracted iEEG data by using a novel three feature encodings technique. They employed a 20 s labeled window, which was processed as the method involved the use of a six Butterworth bandpass filter bank that estimated signal power for all channels. Secondly, the FFT was applied to obtain the frequency spectrum of all channels, also estimating power for all 6 frequency bands. Finally, the cross-channel correlation of all channels was measured by considering only two channels each time. All three features produced 96 feature vectors, except for cross-channel correlation which produced 120 vectors and was used separately. The feature went through SVM classifiers (20 s) where labeled windows were used for training and prediction. This study reported a sensitivity of almost 90–100%, with a false-positive rate of almost 0–0.3 times per day.

Jaiswal and Banka [20] aimed to reduce the computation cost of using wavelet or EMD-based decomposition methods. They used two algorithms that have been used in image processing: the local neighbor descriptive pattern [LNDP] and the one-dimensional local gradient pattern [1D-LGP]) [59]. In the first phase, EEG signals were first transformed into the local pattern, where the technique was used to have the same transformation code for all the similar patterns or structural properties. The second phase was where these transformation codes were used to form the histogram. This is carried out because the distribution of these codes is a convenient form for representing these signal structures. The structural distribution is summarized graphically in two-dimensional spaces by the histogram. The feature vector of the corresponding EEG signal is thus represented by the histogram. Using 10-fold cross-validation ANN, the LNDP and 1D-LGP feature extraction techniques achieved accuracies of 99.82% and 99.80%, respectively.

Kostas et al. [21] used a novel seizure detection technique that avoided complex decision-making processes, training, or empirical boundaries by using rule-based seizure detection logic. This is due to the use of some machine learning techniques that require pre-annotated EEG data or a variant of a priori information during the training process. They utilized EEG segments that contained a high probability of epileptic activity and were automatically isolated for visual inspection and validation. This was achieved without requiring any external intervention and a-priori information. The segments were isolated according to some pre-conditioned criteria. After processing these segments using STFT, the extraction of its EEG signal relative energy distribution among the three energy bands was performed. This method of detecting seizure activation is also another novelty. Instead of using classifiers, signal segments were identified by a clinician according to determined criteria and labeled as segment selection method (SSMx). Only 5%, 3%, or 7% of the prioritized segments were reviewed by a clinician, which means that at least 59% of the data deemed irrelevant was discarded. The results were then reported for the sensitivity and false positive rate of each SSM, for the case of each 5%, 3%, and 7% data discard: The SSM1 had a mean sensitivity of 57%, 69%, and 73% and FPr of 3.1 FP/h, 5.8 FP/h, and 8.4 FP/h, respectively. The SSM II had an improved mean sensitivity of 76%, 80%, and 83%, while the FPr rates reached 4.4 FP/h, 7.3 FP/h, and 10.5 FP/h, respectively. SSM III had a mean sensitivity of 64%, 69%, and 74%, while its FPr rates were 3.9 FP/h, 7.4FP/h, and 10.6 FP/h, respectively. Finally, SSM IV had a mean sensitivity of 84%, 88%, and 92%, while the FPr rate was 4.9 FP/h, 8.1 FP/h, and 12.9 FP/h, respectively.

## 3. Performance Analysis of ML-Based Epileptic Seizure Detection Methods

### 3.1. Performance Evaluation Criteria

In this study, a total of 19 papers were chosen based on their choice of the decomposition and classification methods used to detect seizure signals from EEG. Based on the metrics used, three metrics were chosen for a comparison of the overall performance of the detection system. These are the system’s sensitivity, specificity, and accuracy [37]. Based on these metrics, 10 of the papers were selected since they provided the full metrics while nine papers were rejected. Table A1 in Appendix A provides information for all 19 papers.

The 10 papers were selected to make comparisons between two popular decomposition methods; the wavelet and EMD-based. Out of the ten papers, nine papers were again chosen to make comparisons between the two machine learning classifiers used in these studies, which were the SVM and RF. The EEG signals are usually segmented before being processed by the classifiers. The segments depend on the validation used by the study. Papers that used 10-fold cross-validation used 10 segments of the EEG signals, while five-fold cross-validation used only five segments. These are the labels that indicate if the segments are ictal (seizure), non-ictal (non-seizure), or interictal (the period preceding a seizure segment). The segment’s identifications are labeled as TP, TN, FP, and FN values.

TP: true positive is the identified number of true seizure epochs segments by both algorithm and doctor.
TN: true negative is the identified number of true non-seizure epochs segments by both algorithm and doctor.
FN: false negative is the number of misclassified seizure epochs segments by algorithms, which are recognized as non-seizures, but are actually seizures.
FP: false positive is the number of misclassified seizure epochs segments by algorithms, which are recognized as seizures, but are actually non-seizures.

These parameters are used to calculate the actual parameters used in these studies, shown in the formula below:

(20) $$\mathrm{Sensitivity}=\frac{TP}{TP+FN}\;\times \;100$$

(21) $$\mathrm{Specificity}=\frac{TN}{TN+FP}\;\times \;100$$

(22) $$\mathrm{Selectivity}=\frac{TP}{TP+FP}\;\times \;100$$

(23) $$\mathrm{Accuracy}=\frac{TP+TN}{TN+FP+TP+FN}\;\times \;100$$

By using a simple parameter, such as the mean of all specifications, the overall performance can be evaluated. The evaluation will cover systems that use the popular wavelet and EMD-based decomposition techniques, and another comparison can be made with systems that use the popular RF and SVM-based classifiers.

### 3.2. Wavelet Decomposition Based Seizure Detection

From the 10 studies with full metrics that were selected for comparison, seven of them contained studies that used variants of wavelet-based decomposition of EEG signals, which are displayed in Table 2. Five of the studies used in Table 2 provide a high value of the mean parametric results (above 97%), except for the paper by Jacobs et al. [5] for reasons discussed in the next section. The mean parametric value indicates that the method using the Stockwell transform [9] provided a higher level of system quality in terms of seizure detections at 99.68%. The system’s accuracy was 99.73%, which is also close to the study described in [6]. However, the Stockwell transform’s higher sensitivity value contributes to its better performance. This result was processed using the dataset from The University of Bonn, Germany. For this study, there were three reported results for all parameters; however, the ones mentioned here (i.e., for the ictal state) are the most significant.

It was also observed that the wavelet decomposition method used by Kaleem, Guergachi, and Krishnan [10] also indicated a high level of performance, since all three metrics had higher values by a slight margin. This study used the dataset from CHB-MIT [60]. Both methods used in [9] and [10] employed the k-nearest neighbor for classification; however, in [10] the classifier also used support vector machine together with kNN. Their result was obtained using only 5-fold cross-validation, which also potentially saves processing time. Due to a lack of metrics, it is unfortunate that the quality of some systems could not be evaluated in its entirety, such as the studies described in [12,22] that both had 100% system accuracy but could not be included in this section.

### 3.3. Empirical Mode Decomposition-Based Seizure Detection

From the 10 studies mentioned in Section 3.1, a remainder of three studies provided the mean performance values of the EMD-based seizure detection methods, as shown in Table 3. There were only three studies that were sufficient in the metrics provided to be used in this review. The HVD-based study gave two separate parametric results. However, the HVD-based study [17] stated their parameters by using a range, which makes comparison difficult. Thus, the ranges were separated into their maximum value and minimum values. Their mean parametric performance was at best 98.56% and at worst 96.94%. The study using CEEMDAN [11] also produced two results, because two combinations of all five signals from the dataset were used. Furthermore, the VMD method used in [16] was left out of the comparison table due to the lack of main parameters reported in the study. However, its accuracy of 97.35% was still a good result. It is noteworthy that for the EMD-based decomposition, the classifiers used were restricted to either RF or LS_SVM-based classifiers. All three studies used 10-fold cross-validation on the University of Bonn Dataset from Germany. The comparison indicates that the CEEMDAN decomposition method provides higher system quality. This is for both measurements, which used Set S and (F, N) and Set S and (F) EEG signals at 99.50% and 99% mean parametric value, respectively.

### 3.4. RF Classifier-Based Seizure Detection

From the entire list of 10 eligible papers from Section 3.1, four were used with the same metrics, this time to give a performance comparison of all seizure detection systems that use only RF-based classifiers, as shown in Table 4. Only four studies used the RF classifier and also used different methods of decomposition. Study [11] is presented here with two results due to the combinatorial usage of datasets. Studies [1,5] used the wavelet-based decomposition, while the remaining study [16] used the EMD variant. The VMD method was also left out of the evaluation here due to the lack of the main parameters provided. Table 4 indicates that the CEEMDAN [11] is still the method that provides better overall system performance for seizure detections using the same sets as Table 3.

### 3.5. SVM Classifier-Based Seizure Detection

Five studies with full metrics were chosen from the 10 selected papers for performance comparison of seizure detection methods; however, one which only used SVM classifiers, as shown in Table 5. Five studies are used here with each using the wavelet-based decomposition method, except for [17] which was EMD-based. As explained in Section 3.3, study [17] used the minimum-maximum result, thus giving two results. Considering all studies, the study in paper [10] that used the SVM classifiers and five-fold cross-validation gave an overall performance result of 99.63%. This also means that its metrics are slightly higher than all the other systems in Table 5, except for its specificity which is lower than paper [13]. Coincidentally, both these systems used the wavelet decomposition technique but with a different depth level and order. The other studies in this review either lacked the metrics needed or had metrics which were specified in ranges; thus, they could not be included here.

## 4. Discussion

After the review of 19 articles, 10 papers were used for comparison of their detection of seizure-related signals by EEG. The complete seizure detection system contains multiple-step methods, which are broadly categorized into pre-processing, decomposition, feature extraction, classification, and post-processing. Two prominent decomposition methods used are the wavelet and EMD-based decomposition. Some of the papers used various statistical approaches to decompose the signal. Others used segmentation, such as the method used in [30]. Numerous techniques utilized entropy to detect changes in information power, and/or used it for feature extraction, such as in [61].

Bhattacharyya and Pachori [1] achieved good results in all metrics. Their decomposition method was able to decompose a signal into sub-band frequency while satisfying IMF definitions by the use of an empirical filter. Filters based on these wavelets are adaptive in the sense that they have compact frequency support and are centered around a specific frequency. Dimensions are kept small by employing strategies such as considering the cross-channel interdependence of multivariate data by making use of mutual information, thus reducing the EEG electrodes needed for processing to only three. The best results were obtained using the RF classifier. Further improvements can be made using the naïve Bayes classifier, as it has a “Zero Frequency” problem where if its categorical variable is contained in the test dataset but not in the training dataset, then the model will assign a ‘0’ probability. Some simple smoothing techniques, such as the Laplacian estimation, can be used.

Paper [5] has the advantage of using the complex Morlet wavelet for decomposition, as these types of transform are less oscillatory and are better at detecting and tracking instantaneous frequencies. Another benefit of using this transformed signal includes revealing multiscale frequency information at each time point and isolating noise; in addition, complex Morlet is effective for signal reconstruction. However, its feature extraction technique, called the cross-frequency coupling (IcFc), is based on the concept of thresholding and might be dimensionally burdensome. This method might not be precise as it depends on an RF classifier-based state machine, and could instead be used with other statistical techniques, such as Hjort transform.

Patidar and Panigrahi [6] used a wavelet based on the Daubechies filter with two vanishing moments. Its tunable Q wavelet transform is a filter that is empirical in nature and could provide better time-frequency resolutions. Filters with lower vanishing moments can also be used if they are purposely limited in their ability to decompose signal information adequately without using many resources. This system’s feature extraction stage, which is the Kraskov entropy, uses the Shannon entropy that utilizes a distance function. It is suggested that other distance functions may also be used, such as the Mahalanobis distance function, for improvement together with a two-class discrimination test, such as the Wilcoxon rank test.

The study by Wang et al. [8] used a fifth level wavelet decomposition, which can ensure adequate signal decomposition if five sub-bands are needed with good resource trade-offs. Its dimensions are also kept low at five dimensions. The use of WDTF also improves the selectivity compared to different sub-bands of EEG signals. Its feature extraction method produces a huge vector of 19 × 19 dimensions. By taking the directed transfer function, this dimension is reduced to 19 × 1. Since this dimension calculation is related to energy, using entropies, such as Shannon’s entropy, could improve reduction. Additionally, the use of an elliptic bandpass filter could provide better frequency separation before signal decomposition.

Kalbkhani and Shayesteh [9] used an N-point discrete Fourier transform derivative, which becomes the basis of the Stockwell transform. It provides good resolution in time and frequency. Their use of the nearest neighbor classifier can be further improved with the use of other distance functions, such as the Hausdorff or Mahalanobis.

In the study by Kaleem, Guergachi, and Krishnan [10], the decomposition stage used the level 5 Daubechies db6 wavelet as the mother wavelet with six vanishing moments. A higher number of vanishing moments was used here since it shows more similarity with the recorded EEG signals. However, using the db6 wavelet means more resources are used. The db4 wavelet might characterize the EEG signal without losing information while saving resources for hardware implementation. The use of the dual-tree complex wavelet transform (DT-CWT) by Li, Chen, and Zhang [13] in the decomposition phase is advantageous as it reduces the problem of unreal frequencies, which leads to halfway band separation. They could further improve their system by using the better LS_SVM classifier.

In [11], the authors used a feature extraction stage that had statistical techniques using spectral moments. This technique can be easier to implement on hardware; moreover, it also has better mode mixing separation. In some studies, the authors had a very well-grounded decomposition technique. For example, the main strength of the paper by Zhang, Chen, and Li [16] is in their use of the VMD-based decomposition, which has the capacity to separate two harmonic signals of similar frequency.

Mutlu [17] used the Hilbert vibration decomposition (HVD), which is effective at decomposing both narrow-band and wide-band signals. The use of the LS-SVM classifier is also effective, due to its inequality type constraints. Its pre-processing stage contained a Butterworth low-pass filter that could be replaced with better digital filters, such as the elliptic filters. Parvez and Paul’s [18] use of the undulated global and local feature detection system works on basis of epochs; thus, it has the advantage of being able to process the decomposition more efficiently. For example, its use of phase correlation works by providing shifting information between two correlated signals via Fourier transform. In this method, there is no special decomposition block. However, the window regularization process is complex and could instead be replaced with simpler methods, such as the Hadamard transform. Conversion of time-domain into frequency-domain can also be achieved with other techniques, for example using the Hilbert transform instead of the discrete cosine transform used here.

The study by Ibrahim, Djemal, and Alsuwailem [12] achieved an accuracy of 100% using the discrete wavelet transform with a Rosenstein algorithm that is known to make systems more robust to noise. This system’s next step could be future testing with larger datasets. Khan et al. [7] used a system with only two dimensions and a decomposition technique based on the Mexican hat mother wavelet. Their system has the advantage of a reduction in the trained parameter used in the neural network by using weight sharing. For further improvements, we suggest that other wavelets, such as Coiflet, be used for decomposition as its vanishing point can be fine-tuned. Aside from this, it is also a compactly supported orthogonal wavelet.

Shiao et al.’s paper [19] has a strength where the design choices and performance metrics are closely correlated with clinical objectives. However, their choice of using all channels for feature extraction is costly in terms of resources, since they generated large dimensions for feature extraction (almost 96 to 120 dimensions) despite the fact that they used cross-channel correlation. Techniques such as the various methods of principal component analysis (PCA) can be used for feature dimensions. One such example is the kernel principal component analysis.

Jrad et al. [15] used a seizure detection system where the decomposition of non-stationary signals was precise when dealing with high-frequency oscillation (HFO) signals. Its time-frequency localization was also optimized. The use of Gabor atoms was also advantageous as it was tuned to decompose signals in the physiological band. This system’s classifier could be improved by the use of LS-SVM, since the current RBF kernel used in the SVM classifier has the C parameter that must be properly determined. In their paper, Li, Chen, and Zhang [14] used a wavelet-based envelope analysis (EA) to detect the envelope that was demodulated with Hilbert transform (HT) and to calculate envelope spectrum at each band. Their system was able to reveal the subtle but critical changes contained in EEG signals. Their feature extraction also used a simple statistical measure with low dimensions.

There are also papers with systems that have an advantage in their feature extraction stage. The system from the paper by Jaiswal and Banka [20] has a computationally simple feature extraction that uses a segmentation technique, which means there was better use of resources in the implementation phase. Its use of the ANN classifier is effective; however, the deep CNN is a better classifier. The system described by Tsiouris et al. [21] has the advantage that it uses an unsupervised machine learning classifier that does not require a priori information. In addition, it has an energy-based feature extraction stage, which is easy on hardware resource usage for implementation.

The paper by Goksu [22] is a good example of using a suitable wavelet order for a system. High accuracy is achieved even though only three levels of wavelet decomposition are used, thus saving resources. The entropy used for its feature extraction stage is also simple and allows for extraction of useful information.

## 5. Conclusions

This review attempted to compare and narrow down a large number of available techniques for EEG-based seizure detection and classification based on their superiority of performance. This review focused on the relevant studies on wavelet and empirical mode decomposition-based feature extraction techniques for seizure detection in epileptic EEG data.

The purpose of this review was to compare the seizure detection methods reported recently in several articles. Comparisons of different methods are easily performed when all the standard performance metrics parameters are provided (i.e., sensitivity, specificity, and accuracy). A large number of papers reviewed only gave the accuracy or the false prediction rate, thus limiting the number of papers chosen for full comparison. In this article, the focus is kept on studies that offer full performance metrics.

From the first two comparisons between the systems with wavelet and EMD-based decompositions, it can be concluded that both feature extraction techniques are close contenders; however, the wavelet variant Stockwell transform offers a better detection result in EEG signal-based detection. Almost all extraction techniques compared in this review that delivered good results used five-fold cross-validation classifiers. In conclusion, further research should employ a system using wavelet-based feature extraction technique and five-fold cross-validation technique.

In the last two comparisons, two machine learning classifiers (RF and SVM) were compared on the EEG systems, and it was found that both classifiers had good performance. However, it is noteworthy that the system’s performance also depends on the decomposition method used. This review indicates that the RF classifier works well with EMD-based systems, while the SVM classifier works well on wavelet-based systems.

This review was able to identify the need to choose the relevant combination of decomposition method and classifiers. For future research, this review implies that the correct system combination should be chosen before parameter tuning is performed on any of the classifiers mentioned here for better performance.

## Figures and Tables

**Table 1:** Wavelet transforms used in the selected papers reviewed.

Paper	Wavelet Transform Involved	Characteristics
Bhattacharyya A, Pachori R [1]	Littlewood–Paley and Meyer wavelet	Filters based on these wavelets are adaptive in the sense that they have a compact frequency support and are centered around a specific frequency.
Jacobs D., Hilton T., Del Campo M., et al. [5]	Complex Morlet wavelet	Complex wavelet transform is less oscillatory and is advantageous in detecting and tracking instantaneous frequencies.
Shivnarayan Patidar and Trilochan Panigrahi [6]	Daubechies filter with two vanishing moments	Filters with lower vanishing moments can be used if the filters are purposely limited in their ability to decompose signal information adequately without using many resources.
Wang D, Ren D, Li K, et al. [8]	Daubechies order 4 wavelet Decomposition used up to fifth level	Fifth level decomposition ensures adequate signal decomposition if the user needs an output of five sub-bands with good resource trade-offs.
Hashem Kalbkhani and Mahrokh G. Shayesteh [9]	N-point discrete Fourier transform derivative	This derivative is the basis of the Stockwell transform used by the author. It provides good resolution of time and frequency.
Muhd Kaleem, Aziz Guergachi, and Sridhar Krishnan [10]	Level 5 Daubechies db6 wavelet is used as the mother wavelet with six vanishing moments	The higher number of vanishing moments is used here since it shows more similarity with the recorded EEG signals.
Mingyang Li, Wanzhong Chen, and Tao Zhang [13]	Dual-tree complex wavelet transform (DT-CWT)	Compared to Discrete Wavelet Transform (DWT), the dual-tree types have approximate shift-invariance and preferable anti-aliasing.

**Table 2:** Performance comparison of wavelet-based epileptic seizure detection.

Decomposition Method	Sensitivity (%)	Specificity (%)	Accuracy (%)	Mean Parametric Value (%)	Classifiers	CV
Empirical wavelet transform [1]	97.91	99.57	99.41	98.96	RF	10
CWT with Morlet [5]	87.90	82.40	82.40	84.23	3RF	5
Tunable Q wavelet transform [6]	97.00	99.00	97.75	97.92	LS_SVM	10
Wavelet decomposition (5L-db4) [8]	92.10	99.50	99.40	97.00	RBF_SVM	5
Stockwell transform—ictal [9]	99.42	99.89	99.73	99.68	k-NN	5
Wavelet decomposition (5L-db6) [10]	99.40	99.90	99.60	99.63	SVM	5
Dual-tree complex wavelet transform (DT-CWT) [13]	98.0	100	98	98.6	SVM	10

**Table 3:** Performance comparison of EMD-based seizure detection method for epileptic seizure.

Decomposition Method	Sensitivity%	Specificity %	Accuracy %	Mean Parametric Value (%)	Classifiers	CV
Complete ensemble empirical mode decomposition with adaptive noise [11]-sets S and (F)	100	99	98	99	RF	10
Complete ensemble empirical mode decomposition with adaptive noise [11]-sets S and (F, N)	99.50	100.00	99.00	99.50	RF	10
Variational mode decomposition [16]	-	-	97.35	-	RF	10
Hilbert vibration decomposition [17]	96	97.5	97.33	96.94	LS_SVM	10
	99	99	97.67	98.56	LS_SVM	10

**Table 4:** Performance comparison of seizure detection method using random forest classifiers.

Decomposition Method		Sensitivity %	Specificity %	Accuracy %	Mean Parametric Value	CV
Empirical wavelet transform [1]		97.91	99.57	99.41	98.96	10
CWT with Morlet [5]		87.9	82.4	82.4	84.23	5
Complete ensemble EMD with adaptive noise [11]	S and (F, N)	99.5	100	99	99.50	10
	S and (F)	100	99	98	99.00	10
Variational mode decomposition [16]		-	-	97.532	-	10

**Table 5:** Performance comparison of seizure detection method using SVM-based classifiers.

Decomposition Method	Sensitivity %	Specificity %	Accuracy %	Mean Parametric Value	Classifiers	CV
Tunable Q wavelet transform [6]	97	99	97.75	97.92	LS_SVM	10
Wavelet decomposition (5L-db4) [8]	92.1	99.5	99.4	97.00	RBF_SVM	5
Wavelet decomposition (5L-db6) [10]	99.4	99.9	99.6	99.63	SVM	5
Dual-tree complex wavelet transform (DT-CWT) [13]	98.0	100	98	98.6	SVM	10
Hilbert vibration decomposition [17]	99	99	97.67	98.56	LS_SVM (RBF Kernel)	10
Hilbert vibration decomposition [17]	96	97.5	97.33	96.94	LS_SVM (RBF Kernel)	10

## Data Availability

Publicly available datasets were analyzed in this study. This data can be found here: The CHB-MIT Scalp EEG Database: https://physionet.org/content/chbmit/1.0.0/ (accessed on 5 August 2021). [dataset] Ali Shoeb. 2009. Application of Machine Learning to Epileptic Seizure Onset Detection and Treatment. PhD Thesis, Massachusetts Institute of Technology.

## Appendix A

**Table A1:** List of articles used in this review.

Authors/Dataset Used	Year	Decomposition/Features Used	Classification	Result	Inclusion Criteria
Bhattacharyya A, Pachori R. [1] Dataset: CHB_MIT	2017	Empirical wavelet transform (EWT) Then Hadamard transform removes bias/overfitting to classifiers. Output will be the joint feature vectors.	SMOTE technique used to correct imbalance bias. RF classifier	Max average sensitivity = 97.91% Max average specificity = 99.57% Max average accuracy = 99.41%	Included due to full result specification.
Jacobs D., Hilton T., Del Campo M. et al. [5] Dataset: Toronto Western Hospital Epilepsy Monitoring Unit	2018	IcFc: cross-frequency coupling (CFC) index with a Morlet continuous wave transform	Multi-stage state classifier (MSC) based on three random forest classifiers.	Sensitivity = 87.9% Specificity and accuracy = 82.4%, Area-under-the-ROC (AUC) curve = 93.4%.	Included due to full result specification.
Shivnarayan Patidar. and Trilochan Panigrahi [6] Dataset: University of Bonn Germany	2017	Multi-stage TQWT based decomposition (TQWD) The Kraskov entropy measures and characterizes non-linearities	LS-SVM with RBF kernel functions.	Average accuracy = 97.75% Sensitivity = 97.00% Specificity = 99.00% Matthew’s correlation coefficient = 96.00%.	Included due to full result specification.
Wang D, Ren D, Li K, et al. [8] Dataset: Xi’an Jiaotong University	2018	Wavelet decomposition used with level 5 Daubechies order 4 Directed transfer function	RBF_SVM	Average accuracy = 99.4%, Average selectivity = 91.1%, Average sensitivity = 92.1% Average specificity = 99.5% Average detection rate of 95.8%.	Included due to full result specification.
Hashem Kalbkhani and Mahrokh G. Shayesteh [9] Public Bonn Epilepsy EEG Dataset	2017	Stockwell transform Kernel principal component analysis (KPCA)	Nearest neighbor classifier (kNN)	Ictal (Set E) Sensitivity = 99.42% Specificity = 99.89% Accuracy = 99.73 %	Included due to full result specification.
MuhdKaleem, Aziz Guergachi and Sridhar Krishnan. [10] Dataset: CHB MIT	2017	Level 5 Daubechies db6 wavelet is used as the mother wavelet with six vanishing moments.	Adaptive synthetic sampling (ADASYN) for imbalance problem.	Uses kNN and SVM Sensitivity = 99.8% Specificity = 99.6% Accuracy = 99.6%	Included due to full result specification.
Mingyang Li, Wanzhong Chen and TaoZhang, [13] Public Bonn Epilepsy EEG Dataset	2017	Dual-tree complex wavelet trans-form (DT-CWT)	Wilcoxon test for significance. Support vector machine (SVM)	Accuracy = 98% Sensitivity = 98% Specificity = 100%	Included due to full result specification.
JianJia, Balaji Goparaju, JiangLing Song, et al. [11] Public Bonn Epilepsy EEG Dataset	2017	Complete ensemble empirical mode decomposition with adaptive noise (CEEMDAN)	Random forest classifier Kruskal–Wallis ANOVA	Sets S and (F, N) Accuracy = 99% Sensitivity = 99.5% Specificity = 100% Sets S and (F) Accuracy = 98% Sensitivity =100% Specificity = 99% Cohen’s Kappa statistics = 0.977	Included due to full result specification.
Tao Zhang, Wanzhong Chen and Mingyang Li. [16] Public Bonn Epilepsy EEG Dataset	2017	Variational mode decomposition (VMD) outputs some band-limited intrinsic mode functions (BLIMFs).	Random forest classifier	Highest accuracy is 97.352	Included due to very few papers using EMD-based extraction method, even though no full results.
Ali Yener Mutlu [17] Public Bonn Epilepsy EEG Dataset	2018	Hilbert vibration decomposition (HVD)	(LS-SVM) tested with linear, polynomial and RBF kernel with 10-fold cross-validation	Kernel function/statistical parameters/classification performance (min–max) SPC 95.00–96.50 RBF (= 0.4) ACC 97.33–97.66 SEN 96.00–98.00 SPC 97.5–98.00	Included due to full result specification.
Parvez M, Paul M [18] Dataset: Epilepsy Centre of the University Hospital of Freiburg, Germany	2017	Undulated global feature (UGF) and undulated local feature (ULF) Energy function of CFD (ECFD) and minimum mean energy concentration ratio (MECR) used as feature vector for classification	Least square-SVM classifier with RBF kernel	High prediction accuracy (i.e., 95.4%) FPR = 0.36 Average early prediction time is 22.16 s	Excluded due to no parameter on sensitivity and specificity
Sutrisno Ibrahim, Ridha Djemal and Abdullah Alsuwailem. [12] Dataset: Public Bonn Epilepsy EEG And CHB MIT	2018	Level 6 DWT Daubechies 4 (Db4) Shannon entropy and largest Lyapunov exponent (Rosenstein’s algorithm). Another two conventional methods, which are standard deviation and band power, were also used.	DWT, Shannon entropy, and k-nearest neighbor (KNN) techniques is used. K-NN used with majority vote, K = 3 Linear SVM and LDA	Accuracy = 100%	Excluded due to no parameter on sensitivity and specificity
Khan H, Marcuse L, Fields M, et al. [7] Dataset: The Mount Sinai Epilepsy Center And CHB MIT	2018	Continuous wavelet transform with Mexican mother wavelet. Pre-ictal period and prediction horizon as feature vector. Convolutional neural network used	Deep convolutional neural network used. (stochastic gradient descent with adaptive learning rate). Cross entropy loss function over three classes.	MSSM result Pre-ictal Length = 8 min FPr = 0.128/h CHB MIT result Pre-ictal Length = 6 min FPr = 0.147/h	Excluded due to no parameter on accuracy, sensitivity and specificity
Shiao H, Cherkassky V, Lee J, et al. [19] Dataset: Mayo Clinic	2017	Three feature encodings for iEEG data: Butterworth bandpass filter bank, FFT, and cross-channel correlation of two channels.	Binary SVM classification	Sensitivity = ~ 90–100%, false-positive rate =~ 0–0.3 times per day.	Excluded due to no parameter on accuracy and specificity
Nisrine Jrad, Kachenoura A, Merlet I et al., [15] Dataset: University Hospital of Rennes in France	2016	Convolution of Gabor atom function Gabor root mean square and temporal features Event of interest signals obtained from Gabor RMS	Used RBF- SVM, Receiver operating Characteristic (ROC) curves.	Sensitivity was 0.917 (0.008) for ripples and 0.728 (0.111) for fast ripples while Specificity was 0.738 (0.159) for ripples and 0.933 (0.094) for fast ripples.	Excluded due to no parameter on accuracy
MingyangLi, Wanzhong Chen and TaoZhang. [14] Dataset: Department of Epileptology, University of Bonn	2017	Level 5 Daubechies 4th order discrete wavelet transform. Envelope analysis demodulated with Hilbert transform (HT) for the following extractions: o For the envelope spectrum in each sub-band: mean, energy, standard deviation, and max value. o The mean, energy, standard deviation, and max value of the raw EEG signals.	Neural network ensemble composed of three groups of networks: five sub-nets in each group	Recognition accuracy (RA) = 98.78%	Excluded due to no parameter on sensitivity and specificity
Abeg Kumar Jaiswal, Haider Banka. [20] Dataset:Public Bonn Epilepsy EEG	2017	Local neighbor descriptive Pattern (LNDP) One-dimensional Local Gradient Pattern (1D-LGP)	Artificial neural network classifiers	Average classification accuracy of 99.82% and 99.80%, respectively	Excluded due to no parameter on sensitivity and specificity
Kostas M. Tsiouris, Sofia Markoula, Spiros Konitsiotis et al. [21] CHB MIT	2018	Four novel seizure detection conditions are proposed to isolate EEG segments called Condition I to Condition IV. The short-time Fourier Transform extract EEG energy distribution.	Signal segment used for classifications. All metrics reported here for the case of 3%, 5% and 7% of total visual inspection values respectively.	SSM4 Sensitivity = 84%, 88% and 92% FPr = 4.9 FP/h, 8.1 FP/h and 12.9 FP/h	Excluded due to no parameter on accuracy and specificity
HüseyinGöksu. [22] Public Bonn Epilepsy EEG Dataset	2018	Wavelet packet decomposition. Log energy entropy, norm entropy and energy	Multi-layer perceptron with back propagation	Accuracy = 100%	Excluded due to no parameter on sensitivity and specificity

## Appendix B

**Table A2:** EEG databases and EEG recording techniques.

Paper	Dataset Used	Patients	Recording	Sampling Frequency (Hz)/Resolution (Bit)	Characteristics
1, 7, 10, 12, 21	CHB-MIT	23	9 to 42 records for each patient.	256	Papers using this dataset have used all patient data except paper 7, which used only 22 patients’ data. Only paper 1 and 10 reported using 16-bit resolution in their sampling.
6, 9, 11–14, 16–17, 20, 22	Epilepsy Center of the Bonn University Hospital	5 sets	100 single channel EEG record per subset. 23.6 s each segment.	173.61	5 patient records are available labelled A, B, C, D, and E. However the studies used the following sets: 6,11,17—subset C, D, and E 9,16,20, and 22—used all sets 13 and 14—subset A, D, and E Study 12—subset A vs E Only paper 11 reported using 12-bit resolution in its sampling
5	Toronto Western Hospital Epilepsy Monitoring	12			All patient data were used in this paper; however, the sampling frequency is as below: 500 Hz (5 patients), 512 Hz(3 patients), 1000 Hz (3 patients) and 1024 Hz (1 patients)
7	Mount Sinai Epilepsy Center	28	86 scalp EEG recordings.	256	Only one study used this dataset
8	Institutional Review Boards of Xi’an Jiaotong University	10		200 samples/second	Only one study used this dataset
15	Neurology Department of the University Hospital of Rennes	5		2048	Only one study used this dataset
19	Mayo Clinic	6 (dogs)		400	Only one study used this dataset
